# Immune Portrayal of a New Therapy Targeting Microbiota in an Animal Model of Psoriasis

**DOI:** 10.3390/jpm13111556

**Published:** 2023-10-30

**Authors:** Mihaela Surcel, Carolina Constantin, Adriana Narcisa Munteanu, Diana Antonia Costea, Gheorghița Isvoranu, Elena Codrici, Ionela Daniela Popescu, Cristiana Tănase, Alef Ibram, Monica Neagu

**Affiliations:** 1 Immunology Department, Victor Babes National Institute of Pathology, Splaiul Independentei 99-101, 050096 Bucharest, Romania; mihaela.surcel@ivb.ro (M.S.); adriana.munteanu@ivb.ro (A.N.M.); costea.diana_antonia@s.bio.unibuc.ro (D.A.C.); monica.neagu@ivb.ro (M.N.); 2 Department of Pathology, Colentina University Hospital, Șos. Ștefan cel Mare 19-21, 020125 Bucharest, Romania; 3 Doctoral School of Biology, Faculty of Biology, University of Bucharest, Splaiul Independentei 91-95, 050095 Bucharest, Romania; 4 Animal Husbandry, Victor Babes National Institute of Pathology, Splaiul Independentei 99-101, 050096 Bucharest, Romania; gina.isvoranu@ivb.ro; 5 Biochemistry-Proteomics Department, Victor Babes National Institute of Pathology, Splaiul Independentei 99-101, 050096 Bucharest, Romania; elena.codrici@ivb.ro (E.C.); daniela.popescu@ivb.ro (I.D.P.); 6 Faculty of Medicine, Titu Maiorescu University, Calea Văcăreşti 189, 031593 Bucharest, Romania; bioch@vbabes.ro; 7 Research Laboratory, Romvac Company SA, Şos. Centurii 7, 077190 Voluntari, Romania; alef.ibram@rez.umfcd.ro

**Keywords:** IgY, psoriatic dermatitis, gut–skin axis, cytokines, inflammation

## Abstract

Background: Despite all the available treatments, psoriasis remains incurable; therefore, finding personalized therapies is a continuous challenge. Psoriasis is linked to a gut microbiota imbalance, highlighting the importance of the gut–skin axis and its inflammatory mediators. Restoring this imbalance can open new perspectives in psoriasis therapy. We investigated the effect of purified IgY raised against pathological human bacteria antibiotic-resistant in induced murine psoriatic dermatitis (PSO). Methods: To evaluate the immune portrayal in an imiquimod experimental model, before and after IgY treatment, xMAP array and flow cytometry were used. Results: There were significant changes in IL-1α,β, IL-5, IL-6, IL-9, IL-10, IL-12 (p70), IL-13, IL-15, IL-17a, IFN-γ, TNF-α, IP-10/CXCL10, MCP-1/CCL2, MIP-1α/CCL3, MIP-1β/CCL4, MIG/CXCL9, and KC/CXCL1 serum levels. T (CD3ε^+^), B (CD19^+^) and NK (NK1.1^+^) cells were also quantified. In our model, TNF-α, IL-6, and IL-1β cytokines and CXCL1 chemokine have extremely high circulatory levels in the PSO group. Upon experimental therapy, the cytokine serum values were not different between IgY-treated groups and spontaneously remitted PSO. Conclusions: Using the murine model of psoriatic dermatitis, we show that the orally purified IgY treatment can lead to an improvement in skin lesion healing along with the normalization of cellular and humoral immune parameters.

## 1. Introduction

Psoriasis (PSO) is a typical inflammatory disease with a large clinical involvement of the skin, but it is not restricted to this. Immune cells and their main communication molecules, cytokines/chemokines, circulate systemically; therefore, the inflammatory process is not restricted to the skin-induced dysfunction of various tissues/organs [1]. Inter-cell communication is conducted by a large array of soluble mediators, and as recently shown, cytokines are highly involved with more than 40 different types. As these immune-related molecules drive systemic inflammation, it can affect also the gut system [2]. In PSO, the involvement of the gut–skin axis is supported by in vitro [3] and in vivo studies. In vitro studies have shown that inflammatory dermal pattern affects also gut cells [3]. Studies developed on psoriatic patients have shown that therapies addressing signaling pathways and cytokines can affect also the concurrent diseases of the patient [4]. The gut–skin axis functions both ways, as the imbalanced gut microbiome can modulate the clinical outcome of PSO. Therefore, a recent trial has shown that PSO patients taking probiotics significantly improved their quality of life [5]. A deregulated gut microbiome actually enhances the inflammatory cytokine/chemokine action, driving the progression of PSO [6].

Targeted biologic agents have intensely improved the management of PSO patients, but adverse reactions are still associated with this disease; hence, new approaches or even upgraded classical ones can improve the clinical outcome of this inflammatory disease [7]. Within the new approaches that could be used to improve PSO management, immunoglobulin Y (IgY) has been considered as aiding the panel of adjuvant therapies in both therapeutic and prophylactic regimens. Moreover, oral IgY supplements interact and sustain the microbiome, maintaining the immune system and overall health [8]. 

### Mechanism(s) of IgY Action in the Gastrointestinal Tract

The possible mechanisms of IgY action as a preventive or therapeutic agent in viral, bacterial, or fungal infections have been proposed in the last decade [9]. According to the WHO, IgY antibodies can have activity against 12 bacterial priority pathogens for which novel antibiotics are urgently required [10]. Although avian IgY antibodies have numerous advantages over mammalian IgG for passive immunization [11], at present, the exact mechanism of IgY action in every different pathological challenge still has to be elucidated [12]. Thus, among the possible mechanisms of IgY action, the regulation of the phagocytic capacity of innate immune cells has been proposed to provide passive immunization in mammals [13]. If the beneficial effect of IgY has been observed in numerous experimental pathologic settings [12,14], the exact mechanism of action probably relies on the neutralization of the infectious agent particle. Additionally, IgY administration could alleviate the magnitude of the inflammatory response by modulating the excessive expression of pro-inflammatory cytokines that could extremely damage the organism homeostasis [12,15]. On the other hand, it is known that the IgY molecule possess a structure similarity with the human IgG, the difference being that IgY has four constant regions at the heavy chains by comparison with IgG. This similarity could shape the basis for designing anti-IgY-based therapeutical approaches in different pathological conditions, both for veterinary and human applications [16,17]. 

In terms of oral administration, in passive immunization approaches, IgY has a good structural resistance in the gastrointestinal tract. Thus, one key advantage is that IgY preserves its structure and function at a pH of 3.5–11.0, at a large range of temperature (30–70 °C) and quite stable at the enzyme proteolysis from the gastrointestinal tract [18,19]. However, this molecule’s in vivo properties must be investigated more thoroughly for each species subjected to IgY-based therapy. For instance, IgY antibodies, passing through the gastrointestinal tract of calves, remain biologically functional [9,20]. After IgY enters the gastrointestinal tract and neutralizes the infectious agents against which were generated, the formed complexes follow the clearance of the hepatobiliary route, namely, will leave the organisms through feces as IgY does not address either to the FcR nor to the complement route [19].

Due to its high tolerability, non-allergic characteristics, and no adverse reactions due to the fact that IgY does not link to the Fc receptors or complement system components, it has received attention recently. PSO was found to be associated with a gut-linked deregulated microbiota [21,22] and this gut dysbiosis can influence the skin microbiota and induce the enhancement in psoriatic events [23]. In our previously published PSO animal model, we showed that IgY raised against pathological human antibiotic-resistant bacteria can ease psoriatic lesions and restore the deregulated immune cell parameters induced by the psoriatic events [24].

**Figure 1:**
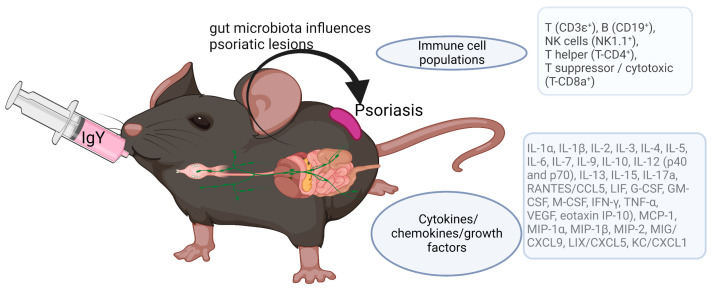
Parameters studied in the experimental murine model. IgY raised against pathological human bacteria resistant to antibiotics were orally administered in PSO-C57 BL/6 mice; cytokine/chemokine serum levels were followed in relation to the circulatory immune cell populations.

The studied murine model (Figure 1) evaluated the orally applied IgY treatment effect in psoriasiform dermatitis induced by imiquimod (IMQ). Mice were followed for the circulatory cytokine/chemokine profile in relation to the immune cell populations.

Following our endeavor to elucidate in depth the immune mechanisms that govern cell–cell communications, in this paper, we test in our psoriatic IMQ-induced mouse model 32 cytokines/chemokines involved in the main immune functions appending to PSO and to the experimental therapy with specific IgY triggering the gut microbiota.

## 2. Materials and Methods

### 2.1. Isolation and Purification of IgY

The IgY used in this study was an original product of the ROMVAC Company SA and is part of the IMUNOINSTANT brand, with a European trademark (EUIPO). IgY was isolated by ROMVAC Company SA from the yolk of hyperimmune eggs from hens immunized with a mixture of human pathogenic antigens, according to the methodology described in the patent [12]. These pathogens (*Acinetobacter baumannii*, *Clostridium difficile*, *Enterococcus faecalis*, *Escherichia coli*, *Klebsiella pneumonia*, *Salmonella* spp., and *Streptococcus pneumonia*) were selected due to their antibiotic-resistant characteristics and are highly involved in nosocomial infections [25].

After the standard isolation protocol of IgY, the compound was purified and isolated using next-generation chromatography (NGC Chromatography System, Bio-Rad, Hercules, CA, USA), according to the manufacturer’s instructions. A high-resolution column size exclusion ENrich SEC 650 (Bio-Rad, Hercules, CA, USA) was used in order to separate the biomolecules with molecular weights of 500 Da–650 KDa. v chromatographic analysis involved the following steps: column equilibration and calibration, standard/sample injection, and standard/sample elution. Column equilibration was performed with 2 column volumes (CV, 50 mL) of phosphate-buffered saline (PBS tablets, 100 mL, VWR Life Science, Columbus, OH, USA), and the column was considered equilibrated when no changes in pH were recorded at a minimum 5 mL run volume. All buffers were previously filtered (Nalgene Rapid-Flow 75 mm Filter Units 500 mL, Thermo Scientific, Waltham, MA, USA; and Vacuum pump, Merck, Millipore, NY, USA). In order to calibrate the column, a lyophilized mixture of thyroglobulin (Mr 670,000 Da), bovine γ-globulin (Mr 158,000 Da), chicken ovalbumin (Mr 44,000 Da), equine myoglobin (Mr 17,000 Da), and vitamin B12 (Mr 1350 Da) (Gel Filtration Standard, Bio-Rad, Hercules, CA, USA) was used. Standards and samples (1 mL) were introduced in the NGC system through an injection valve, after being previously filtered (Puradisc 25PP, Whatman, Maidstone, UK). We also used standard IgY (chicken IgY DEAE purified from mixed-breed chicken eggs, 10 mg/mL, Lampire Biological Laboratories, Pipersville, PA, USA). Standards and samples were eluted with a single volume of PBS at a flow rate of 1 mL/min (the optimal flow rate of the ENrich SEC 650 column is 0.75–1.25 mL/min), pressure of 233 PSI, absorbance of 280 nm, and pH = 8. Each chromatographic peak was pooled by repeated injection with the corresponding test tube through the fraction collector (BioFrac Fraction Collector, Bio-Rad, Hercules, CA, USA). Additional technical details are to be found in the filled patent [26].

### 2.2. IMQ-Based Murine Model

The experimental model used C57 BL/6 mice (Jackson Laboratory, Bar Harbor, ME, USA), males and females (10–11 weeks), bred by the Animal Husbandry of Victor Babeș National Institute of Pathology, Bucharest, Romania. Mice were provided with optimal housing conditions (temperature 22 ± 2 °C, humidity 55 ± 10%, artificial ventilation, 12/12 light/dark cycle). The animals were kept in open cage systems (food and water ad libitum) and were monitored daily. The experiments were conducted in accordance with recognized principles of laboratory animal care according to EU Directive 2010/63/EU 45 and the experimental study was approved by the Victor Babeș Institute’s Ethics Committee (88/20 January 2021) and National Sanitary Veterinary and Food Safety Authority (598/08 February 2021)

The experimental IMQ mice model of psoriatic dermatitis was performed according to protocols as previously described [27,28]. Two groups of C57BL/6 mice were studied (1:1 sex ratio), of 10–11 weeks old, with a mean weight of 21 ± 3 g (Balance Scientech SL-3100D; Boulder, CO, USA), free of any medication in the previous 72 h: (i) the psoriasis group (PSO) (36 mice) that received a daily topical dose of 62.5 mg IMQ cream (5% Aldara Cream; Meda AB, Sweden) on the shaved back for 6 consecutive days (a daily dose of 3.125 mg of active compound); and (ii) the normal group (Control) (8 mice) with no topical treatment that received the same volume sterile PBS through gavage in parallel with the treatment groups.

The PSO-induced group of C57 BL/6 mice was divided into the following groups:

- PSO group (PSO) (8 mice—1:1 sex ratio) with induced psoriatic dermatitis as described above without treatment. The mice were sacrificed on day 7 of the experiment.
- IgY-treated PSO group (PSO IgY) (12 mice—1:1 sex ratio) with induced psoriatic dermatitis received (starting with day 7) a gavage dose of 37.5 µg IgY in a volume of 50 μL sterile PBS, for 5 consecutive days; the dose matched the dose of IgY given to a human adult (g/kg) according to a study case [29]. Mice were sacrificed on day 20 when the psoriatic lesions were macroscopically remitted.
- Purified IgY-treated PSO group (PSO PIgY) (8 mice—1:1 sex ratio) with induced psoriatic dermatitis, received the same dose of IgY but with the purified one in the same volume and were sacrificed on day 20 as above.
- Naturally remitted PSO group (Remitted PSO) (8 mice—1:1 sex ratio) with induced psoriatic dermatitis received (starting with day 7) a gavage with a volume of 50 μL sterile PBS for 5 consecutive days and then were allowed to heal naturally and sacrificed on day 22 when natural remission was assessed macroscopically.

**Figure 2:**
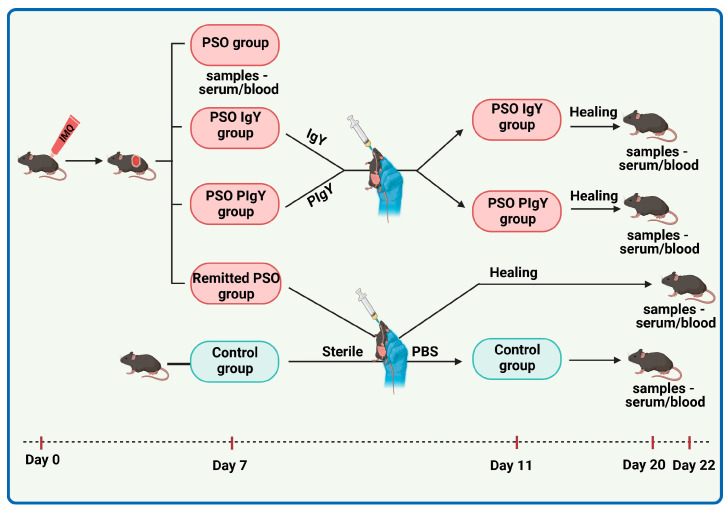
The study design over time. The graphic scheme of the study includes the main key points of the experiment: induction of murine PSO with IMQ (Day 0–Day 6), oral administration of IgY/PIgY treatment by gavage (Day 7–Day 11), healing period (up to Days 20/22), and harvesting of biological samples (Days 7, 20, and 22).

An outline of the experimental model, timeline, and procedures is presented in Figure 2.

Clinically, the mice groups were scored using PASI evaluation, as previously detailed by us [11]. Briefly, skin inflammation was assessed using in vivo measurements (erythema, desquamation, and induration parameters—EDI) and PASI score. EDI were monitored daily on a 0–4 scale (0—none; 1—slight; 2—moderate; 3—marked; and 4—very marked) and a modified PASI score (erythema + desquamation + induration) was calculated daily on a 0–12 scale in order to score the inflammation due to IMQ–PSO induction, natural remission, and IgY treatment. 

Samples. In order to harvest blood, spleen, and skin samples at the end of the experiments, the animals received standard anesthesia with a ketamine/acepromazine/xylazine cocktail (ketamine 80 mg/kg, Richterpharma ag, Austria; acepromazine 6 mg/kg, Vetoquinol SA, Lure, France; xylazine 1 mg/kg, Bioveta SA, Czech Republic). Peripheral blood was collected by intra-cardiac puncture in K2-EDTA-coated tubes (Microvette, Sarsted AG & Co. Nümbrecht, Germany) and in anticoagulant-free tubes for serum harvesting. For splenomegaly evaluation, the individual spleens were weighed (Balance AEP-1500A, Adam Equipment Co., Ltd., Kingston, UK). Skin samples were collected and processed (fixed in 10% buffered formalin, embedded in paraffin, and sectioned in 5 µm sections) for hematoxylin and eosin (H&E) staining, prior to histopathological evaluation (Olympus BX43 with CellSens Dimension Program, Tokyo, Japan).

### 2.3. Serum Cytokines Testing Using xMAP Array Analysis

Luminex200 equipment (Luminex Corp., Austin, TX, USA) and MILLIPLEX MAP Mouse Cytokine/Chemokine Magnetic Bead Panel Kit (Millipore, Burlington, MA, USA) were used to quantify the levels of serum cytokines, following manufacturer’s settings and guidelines. The kit allows the simultaneously analysis of 32 cytokines/chemokines: interleukin (IL)-1α, IL-1β, IL-2, IL-3, IL-4, IL-5, IL-6, IL-7, IL-9, IL-10, IL-12 (p40), IL-12 (p70), IL-13, IL-15, IL-17a, RANTES (regulated upon activation, normal T-cell levels expressed and presumably secreted)/CCL5, leukemia inhibitory factor (LIF), granulocyte colony-stimulating factor (G-CSF), granulocyte–macrophage colony-stimulating factor (GM-CSF), macrophage colony-stimulating factor (M-CSF), interferon (IFN)-γ, tumor necrosis factor (TNF)-α, vascular endothelial growth factor (VEGF), eotaxin, IFN-γ-inducible protein-10 (IP-10), monocyte chemoattractant protein (MCP)-1, macrophage inflammatory protein (MIP)-1α, MIP-1β, MIP-2, monokine induced by gamma interferon (MIG/CXCL9), lipopolysaccharide-induced CXC chemokine (LIX/CXCL5), and keratinocyte-derived chemokine (KC/CXCL1). According to the producer’s guidelines, the beads were incubated (4 °C overnight and shaking at 800 rpm) with buffer, cytokine standards, and samples in a 96-well plate, after the first incubation, followed by beads, detection antibodies, and streptavidin phycoerythrin conjugate (SAPE) (at room temperature, in the dark, and shaking) incubation. Multiplex data acquisition and analysis was performed using the Luminex200 platform provided with the xPONENT 4.2 software (Luminex Corp., Austin, TX, USA). Duplicate samples were used for all specimens and the calibration curves were generated with a 5-parameter logistic fit. Results are presented as mean ± SD of pg/mL serum concentrations.

### 2.4. Flow Cytometry Analysis

Flow cytometry (BD FACSCanto II cytometer, BD Biosciences, San Jose, CA, USA) was performed in order to quantify the main lymphocyte populations: T (CD3ε^+^), B (CD19^+^), and NK cells (NK1.1^+^). T-helper (T-CD4^+^) and T-suppressor/cytotoxic (T-CD8a^+^) subsets were also assessed, and the T-CD4^+^/T-CD8^+^ ratio was calculated. Peripheral blood was incubated with TruStain fcX (anti-mouse CD16/32, isotype Rat IgG2a, λ) antibody (BioLegend, San Diego, CA, USA) for 7 min on ice, and then stained for 20 min at room temperature and in the dark with the following monoclonal antibodies: 0.5 µL Alexa Fluor 647 anti-mouse CD3ε (clone 145-2C11, isotype Armenian Hamster IgG); 0.5 µL Alexa Fluor 488 anti-mouse CD8a (clone 53–6.7, isotype Rat IgG2a, κ); 1.25 µL PE-Cy7 anti-mouse CD4 (clone GK1.5, isotype Rat IgG2b, κ); 1.25 µL PerCP-Cy5.5 anti-mouse CD19 (clone 6D5, isotype Rat IgG2a, κ); and 1.25 µL PE anti-mouse NK1.1 (clone PK136, isotype Mouse IgG2a, κ) (all from BioLegend, San Diego, CA, USA). After red blood cell lysis (BD FACS Lysing Solution, BD Biosciences, San Jose, CA, USA) and two washing steps with cell-staining buffer, the samples were acquired and analyzed with a flow cytometer using the BD FACSDiva v 6.1 software (BD Biosciences, San Jose, CA, USA). The compensation of spectral overlaps was performed using UltraComp eBeads (Invitrogen by Thermo Fischer Scientific, San Diego, CA, USA), and the BD Cytometer Setup & Tracking Beads Kit (BD Biosciences, San Jose, CA, USA) was used for the daily check up of the cytometer performance. T-CD4^+^ and T-CD8a^+^ were expressed as percentages of CD3ε^+^ lymphocytes, and B and NK cells as percentages of CD3ε^-^ lymphocytes.

### 2.5. Statistical Analysis

GraphPad Prism 9.4.0 (GraphPad Software, San Diego, CA, USA) and Microsoft Excel (Version 2310) (Microsoft, Redmond, WA, USA) were used, and results are expressed as mean values ± standard deviation (SD). The data were processed using an ordinary one-way analysis of variance (ANOVA) with the application of Tukey’s multiple comparison test, and adjusted *p*-values were considered. The levels of statistical significance were considered at *p* < 0.05. Pearson’ correlation coefficient (−1 < r <1) was used to determine the correlation between the parameters; for an r-value close to ±1, the correlation was considered strong (positive/negative).

## 3. Results

### 3.1. NGC-Purified IgY

**Figure 3:**
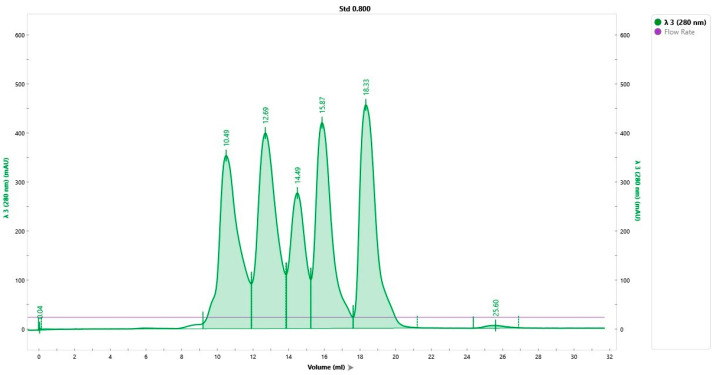
Gel filtration standard chromatogram for the optimization of sample preparation. Detection at 280 nm of thyroglobulin (Mr 670,000 Da), bovine γ-globulin (Mr 158,000 Da), chicken ovalbumin (Mr 44,000 Da), equine myoglobin (Mr 17,000 Da), and vitamin B12 (Mr 1350 Da).

In order to purify the IgY obtained from the yolk of hyperimmune eggs, we used NGC calibrated with the gel filtration standard (Bio-Rad, Hercules, CA, USA). The standard was eluted with 1 CV (25 mL) of PBS at a flow rate of 1 mL/min, 233 PSI, absorbance at 280 nm, and pH of 8. The chromatogram for the gel filtration standard is presented in Figure 3.

**Figure 4:**
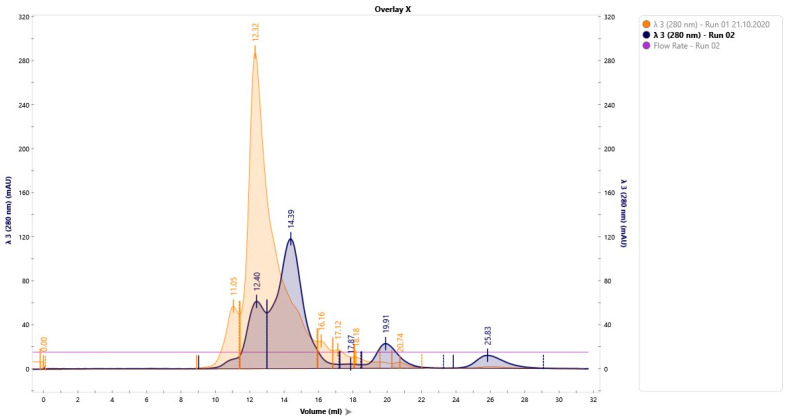
IgY standard’s and IgY sample’s overlapped chromatograms. The IgY standard (yellow) and IgY sample chromatograms (blue) were obtained at a flow rate of 1 mL/min, 233 PSI, absorbance at 280 nm, and pH of 8.

After the column calibration, the standard IgY and samples were further eluted in the same conditions (1 mL/min flow rate, 233 PSI, 280 nm, and pH of 8). To compare the purification flow, we overlapped the chromatograms obtained for the standard IgY and IgY sample, as shown in Figure 4.

Following the overlapping of the two chromatograms, four peaks were identified (Figure 2, colored in blue). Protein fractions corresponding to these peaks were collected and the concentrations were determined using a Nanodrop 2000 spectrophotometer (ThermoFisher Scientific, Waltham, MA, USA) (Table 1).

Protein fractions corresponding to peaks 1 and 2 were mixed and used in the experimental model as purified IgY (PIgY).

### 3.2. IMQ-Induced Inflammation and IgY-Induced Healing

As previously established, IMQ induces a severe of skin inflammation, and animals were scored as PSO patients using individual PASI scores, namely, erythema, thickening, and skin scaling. Moreover, at the end of experiment, splenomegaly evaluation and histopathological assessment were performed as previously published [11]. Clinically, the mice treated with the NGC-purified IgY had the same follow-up as the IgY-treated mice and were euthanized at day 20, when lesions were macroscopically remitted for both groups. All data gathered for the NGC-purified IgY for splenomegaly evaluation and histopathological assessment proved to be similar to that of the controls, as described by us for the IgY-treated mice [24].

**Figure 5:**
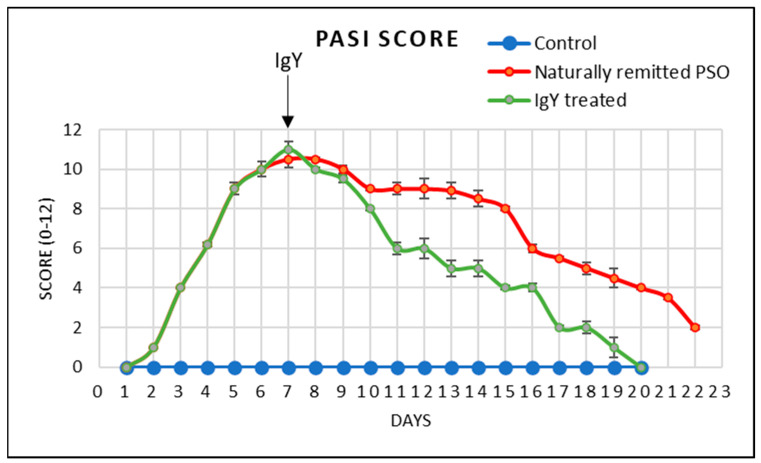
PASI score in the control, in the naturally remitted PSO, and the IgY-treated groups (mean ± SD).

Clinically, out of all EDI parameters, erythema is the first parameter that can be scored after one day of IMQ application, followed after another day by the subsequent registered parameters; thus, starting from day 2, all EDI parameters are registered in all the animals subjected to IMQ, as previously shown by us [11]. Similarly to PSO patients, in our animal model, the severity of inflammation was estimated based on a modified PASI score (0–12 scale), calculated daily by adding the independent daily scores obtained for EDI (in our experimental model, the affected area was not taken into account as the affected surface skin area was identical to all mice). The PASI score had a progressive evolution during the IMQ treatment (Figure 5), matching the increased severity of the psoriatic lesions. In time, there were some individual mice in the naturally remitted PSO group that had a score of 2 at day 22, while the IgY-treated mice had a decrease in the PASI score at day 19 and their skin did not display any PASI score. This clinical evaluation of the experimental group with and without IgY treatment was also confirmed by us in a previous publication [24]. The decrease in the PASI score was statistically significant in the IgY-treated group starting from day 10 (Table 2). Hence, after the first 3 days of therapy, it continued to decrease abruptly in comparison to the naturally remitted group.

### 3.3. Evaluation of the Serum Cytokine/Chemokine Profile in the IgY-Treated Mice

An xMAP array analysis was used in order to evaluate the immunological changes in circulatory cytokines/chemokines upon the IgY treatment of IMQ-induced experimental PSO. Among the 32 investigated cytokines/chemokines/growth factors, significant changes in circulatory levels were obtained for IL-1α, IL-1β, IL-5, IL-6, IL-9, IL-10, IL-12 (p70), IL-13, IL-15, IL-17a, IFN-γ, TNF-α, IP-10/CXCL10, MCP-1/CCL2, MIP-1α/CCL3, MIP-1β/CCL4, MIG/CXCL9, and KC/CXCL1. We further present the individual cytokines/chemokines and their immunological relevance in terms of PSO pathogenesis.

#### 3.3.1. IL-12 (p70) and IL-6 Circulatory Levels

**Figure 6:**
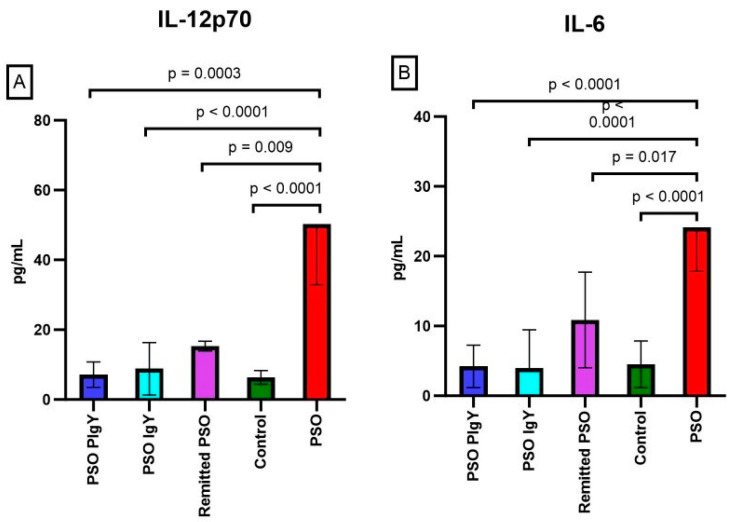
Serum levels of IL-12 (p70) and IL-6. (**A**). IL-12 (p70) in the PSO PIgY group (7.1 ± 3.6), PSO IgY group (8.8 ± 7.5), remitted PSO group (15.3 ± 1.4), control (6.3 ± 2.0), and untreated PSO group (50.3 ± 17.4); (**B**). IL-6 in the PSO PIgY group (4.2 ± 3.0), PSO IgY group (3.9 ± 5.5), remitted PSO group (10.8 ± 6.8), control (4.5 ± 3.3), and untreated PSO group (24.1 ± 6.2). The results are presented as the mean concentration values ± SD.

IL-12 and IL-6 are important regulators of Th1/Th2 differentiation. IL-6 also mediates the differentiation of Th17 cells in the presence of IL-23. In the experimental model of psoriatic dermatitis, the evaluation of IL-12 (p70) and IL-6 cytokines values showed statistically significant differences among all the investigated groups (Figure 6).

The serum levels of IL-12 (p70) and IL-6 were highly increased in the IMQ-treated mice (PSO group) (50.3 ± 17.4; 24.1 ± 6.2) compared to the controls (6.3 ± 2.0; 4.5 ± 3.3) (*p* < 0.0001) (Figure 6A,B). Significant differences were observed among the values obtained for the PSO group, IgY-treated groups, and the remitted PSO group. After IgY treatments, we observed the normalization of these values, especially for the PSO PIgY group, where the expression level of these cytokines matched the ones found in the controls (7.1 ± 3.6; 4.2 ± 3.0 vs. 6.3 ± 2.0; 4.5 ± 3.3). Furthermore, for both cytokines, in both the PSO PIgY and PSO IgY groups, the values matched those of the control groups (*p* > 0.05), underlining the normalization of these values after IgY treatment. For the naturally healing group (remitted PSO group), the values of these modulators also decreased at the end of experiment, but there were still statistical significant differences compared to those of the controls for IL-12 (p70) (*p* = 0.0001). The normalization of the values was more evident in the case of IgY treatments. We obtained a strong negative correlation (r = −0.527) between IL-6 and IL-12 (p70) in the PSO group.

#### 3.3.2. Circulatory Levels of TNF-α and IFN-γ

**Figure 7:**
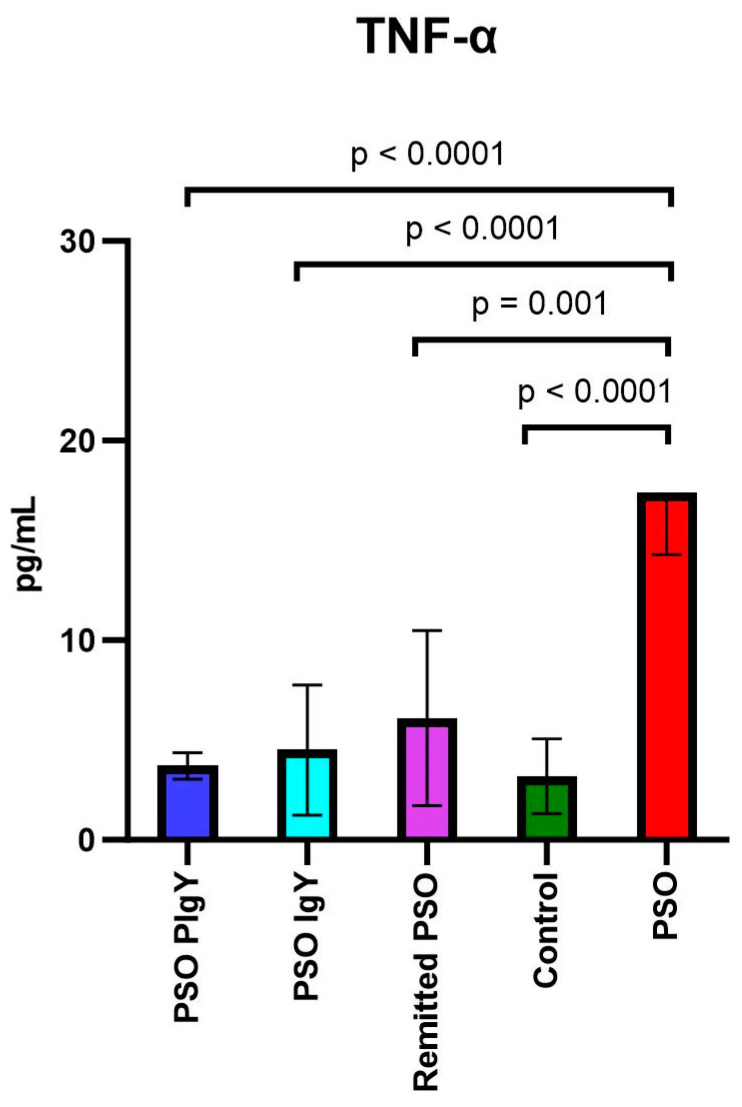
Serum levels of TNF-α. TNF-α in the PSO PIgY group (3.7 ± 0.7), PSO IgY group (4.5 ± 3.3), remitted PSO group (6.1 ± 4.4), control (3.2 ± 1.9), and untreated PSO group (17.4 ± 3.1). The results are presented as the mean concentration values ± SD.

The evaluation of Th1-specific cytokines revealed statistically significant differences between the experimental groups for TNF-α, while IFN-γ presented detectable values only in the PSO mice (Figure 5). As in the case of IL-12 and IL-6, the serum levels TNF-α were highly increased in the IMQ mice (PSO group) (17.4 ± 3.1) compared to those of the controls (3.2 ± 1.9) (*p* < 0.0001) (Figure 7). 

The statistical analysis showed significant differences among the PSO group, IgY-treated groups, and the remitted PSO group. We registered the normalization of these values after IgY treatments, mainly for the mice treated with purified IgY, where the circulatory level of this cytokine was similar to that of the controls (3.7 ± 0.7 vs. 3.2 ± 1.9). In the PSO PIgY and PSO IgY groups, the levels of the investigated cytokines were identical to those of the control group (*p* > 0.05), reinforcing the normalization of these values after IgY treatment. For the naturally healing group, the value of TNF-α was also found to be decreased at the end of experiment, similar to the values obtained for the controls. The analysis of the serum IFN-γ level revealed detectable values only for IMQ mice (PSO group) (45 ± 6.2) and a strong negative correlation between IFN-γ and IL-12 (p70) in the PSO group was noticed (r = −0.805).

#### 3.3.3. Circulatory Levels of IL-1α and IL-1β

**Figure 8:**
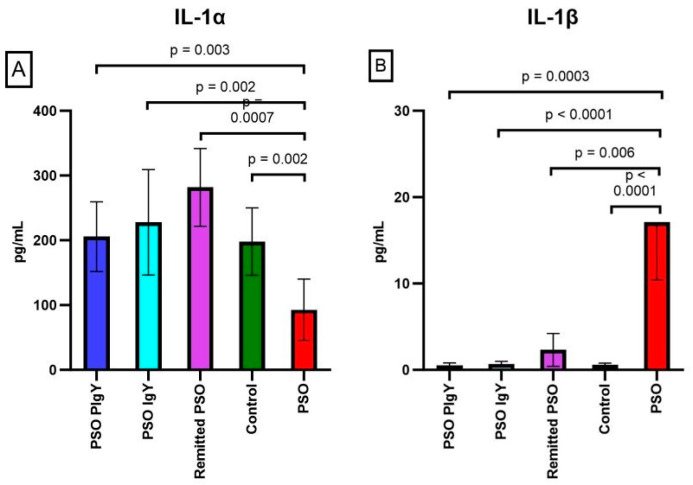
Serum levels of IL-1α and IL-1β. (**A**) IL-1α in the PSO PIgY group (205.8 ± 53.9), PSO IgY group (227.8 ± 81.3), remitted PSO group (281.8 ± 59.9), control (198.1 ± 51.9), and PSO group (92.8 ± 47.5). (**B**) IL-1β in the PSO PIgY group (0.5 ± 0.3), PSO IgY group (0.7 ± 0.3), remitted PSO group (2.3 ± 1.9), control (0.6 ± 0.2), and PSO group (17.1 ± 6.7). The results are presented as the mean concentration values ± SD.

In the PSO mice, IL-1α presented significantly decreased mean values compared to those of the controls (92.8 ± 47.5 vs. 198.1 ± 51.9, *p* = 0.002) (Figure 8A). The serum levels of IL-1α in the PSO group were strongly positively correlated to TNF-α values (r = 0.611) and negatively correlated to IL-1β (r = −0.495). After IgY treatments and natural healing, we observed a pronounced upward trend for all experimental groups (all *p*-values were lower than 0.05), especially for the remitted PSO group (281.8 ± 59.9). Thus, IgY treatments led to the normalization of IL-1α serum values (205.8 ± 53.9 for PSO PIgY and 227.8 ± 81.3 for PSO IgY groups), statistically matching the control values (*p* > 0.05). This result strengthens the finding that the IgY treatment leads to the normalization of the investigated parameters. The serum levels of IL-1β were found highly increased in IMQ mice (PSO group) (17.1 ± 6.7) compared to those of the controls (0.6 ± 0.2) (*p* < 0.0001) (Figure 8B).

The statistical analysis showed significant differences among the PSO group, IgY-treated groups, and the naturally remitted PSO group. After IgY treatments, we observed the normalization of these values, especially for the PSO PIgY group, where the circulatory level of these cytokines were low as the control group levels (0.5 ± 0.3 vs. 0.6 ± 0.2, respectively). As in the already mentioned cytokines, in the PSO PIgY and PSO IgY groups, the registered levels matched the ones of the control group (*p* > 0.05), showing the normalization of these values after IgY treatment. For natural healing (remitted PSO group), the values of IL-1β were also found to be decreased at the end of experiment, but interestingly, the values were still statistically significantly different compared to those of the controls (*p* = 0.03). We obtained a strong positive correlation (r = 0.563) between IL-1β and IL-12 (p70) in the PSO group and also in the purified IgY-treated (r = 0.534) PSO group. Strong negative correlations between IL-1β and IFN-γ in the PSO group were noticed (r = −0.494) as well.

#### 3.3.4. Serum Levels of IL-9, IL-15, and IL-17

**Figure 9:**
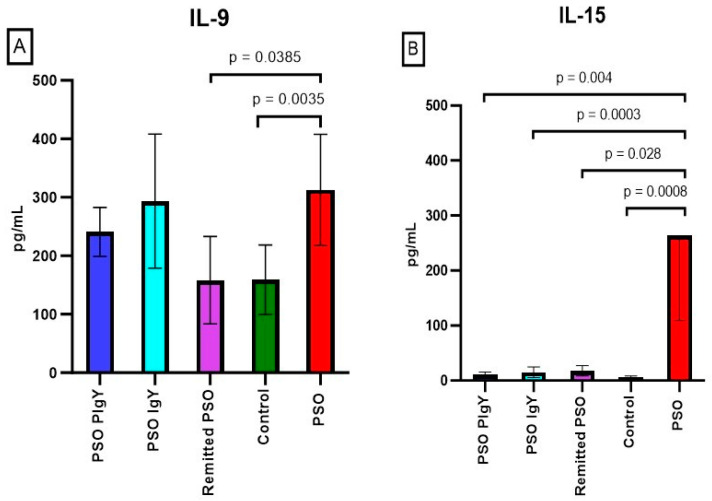
Serum levels of IL-9 and IL-15. (**A**) IL-9 in the PSO-PIgY group (241.0 ± 41.9), PSO IgY group (293.5 ± 114.5), remitted PSO group (158.3 ± 74.8), control (159.1 ± 59.3), and the untreated PSO group (312.5 ± 95.0). (**B**) IL-15 in the PSO PIgY group (10.5 ± 4.9), PSO IgY group (15.2 ± 9.7), remitted PSO group (17.9 ± 9.3), control (5.8 ± 2.5), and untreated PSO group (263.6 ± 154.4). The results are presented as the mean concentration values ± SD.

IL-9 and IL-15 presented significant elevated circulatory levels in IMQ mice compared to those of the controls (312.5 ± 95.0 vs. 159.1 ± 59.3, *p* = 0.0035; and 263.6 ± 154.4 vs. 5.8 ± 2.5, *p* = 0.0008, respectively) (Figure 9A,B).

A strong negative correlation (r = −0.559) was also noticed between these molecules in the PSO group. In the case of IL-9, even though a decreasing trend was observed after IgY treatments, the values still remain significantly higher than in controls (241.0 ± 41.9, *p* = 0.0248 for PSO-PIgY and 293.5 ± 114.5, *p* = 0.0139 for PSO-IgY). For the naturally healed mice, the mean values of serum IL-9 were similar to those of the controls. The serum levels of IL-9 in the PSO group were strongly positively correlated to IL-6 (r = 0.692) values and negatively correlated to IL-12 (r = −0.480) levels. The serum level of IL-15 decreased significantly after both IgY treatments and natural healing, but in the remitted PSO group, IL-15 still remained high compared to that of the controls. A strong positive (r = 0.474) correlation was obtained between IL-15 and IFN-γ in the IMQ mice (PSO group).

As expected, the IL-17 circulatory level was found to be higher in IMQ mice compared to that of the controls (28.0 ± 26.5 vs. 5.8 ± 2.2, *p* = 0.049) and lowered in the IgY-treated groups (4.4 ± 2.0 for PSO PIgY and 4.9 ± 4.2 for PSO IgY) and in the naturally remitted PSO group (6.9 ± 4.5). The analysis of IL-17 serum values revealed statistically significant differences between the males from the PSO group and the control males (53.6 ± 19.2 vs. 6.6 ± 1.6, *p* = 0.0258), differences that were not observed in females nor in the other experimental groups. The serum levels of IL-17 in the PSO group were strongly positively correlated to IL-15 (r = 0.572) values and negatively correlated to IL-1α (r = −0.659), IL-9 (r = −0.642), and TNF-α (r = −0.518) levels.

#### 3.3.5. Circulatory Levels of IL-5, IL-10, and IL-13

**Figure 10:**
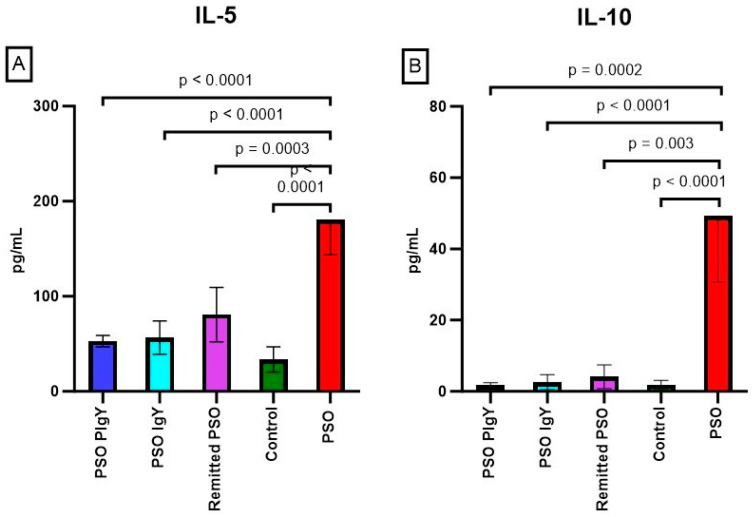
Serum levels of IL-5 and IL-10. (**A**) IL-5 in the PSO PIgY group (52.9 ± 6.0), PSO IgY group (56.5 ± 17.5), remitted PSO group (80.7 ± 28.6), control (33.7 ± 13.3), and untreated PSO group (180.3 ± 36.5). (**B**) IL-10 in the PSO PIgY group (1.7 ± 0.7), PSO IgY group (2.6 ± 2.1), remitted PSO group (4.1 ± 3.3), control (1.8 ± 1.3), and untreated PSO group (49.3 ± 18.7). The results are presented as the mean concentration values ± SD.

The profile of IL-5 and IL-10, which are Th2-specific cytokines, was found to be improved after the IgY treatment. As in the other cases, the mean concentration values obtained for the PSO group were statistically different (Figure 10). The IL-5 and IL-10 serum levels had elevated values in the PSO group compared to those of the controls (*p* < 0.0001); moreover, in the IgY-treated groups and in the naturally remitted PSO group, the cytokine levels dropped in a statistically significant way, with all *p*-values being lower than 0.05 (Figure 10A,B).

The serum levels of IL-5 in the PSO group were strongly positively correlated to TNF-α values (r = 0.754) and negatively correlated to IFN-γ (r = −0.695) levels. Strong positive and negative correlations (r = 0.612 and r = −0.660, respectively) were obtained among IL-5, IL-6, and IL-10 in the PSO group. IgY treatments led to a decreased secretion of these cytokines, and for IL-10, the levels were similar to those of the controls (*p* > 0.05). Even though the serum level of IL-5 decreased significantly after IgY treatment, the values still remained higher compared to those of the controls (*p* = 0.0134 for the PSO PIgY group; *p* = 0.0126 for PSO IgY). In the group treated with purified IgY, strong positive and negative correlations (r = 0.529 and r = −0.879, respectively) were also observed among IL-5, IL-6, and IL-10. For the naturally healing group, we also obtained decreased mean values, but the normalization of the values was more obvious in the case of IgY treatments. The serum level of IL-13, also a Th2-specific cytokine, showed significant decreased values in IMQ mice compared to that of the controls (59.1 ± 17.4 vs. 124.0 ± 72.2, respectively; *p* = 0.0394), and these values decreased after IgY treatments and natural healing (39.9 ± 12.0 for the PSO PIgY group, 38.0 ± 13.0 for the PSO IgY group, and 55.2 ± 20.9 for the naturally remitted group). A strong positive correlation (r = 0.806) was noticed between IL-13 and IL-10, and a negative one between IL-13 and IL-1β in the IMQ mice (PSO group).

#### 3.3.6. Serum Levels of MCP-1/CCL2, MIP-1α/CCL3, and MIP-1β/CCL4

**Figure 11:**
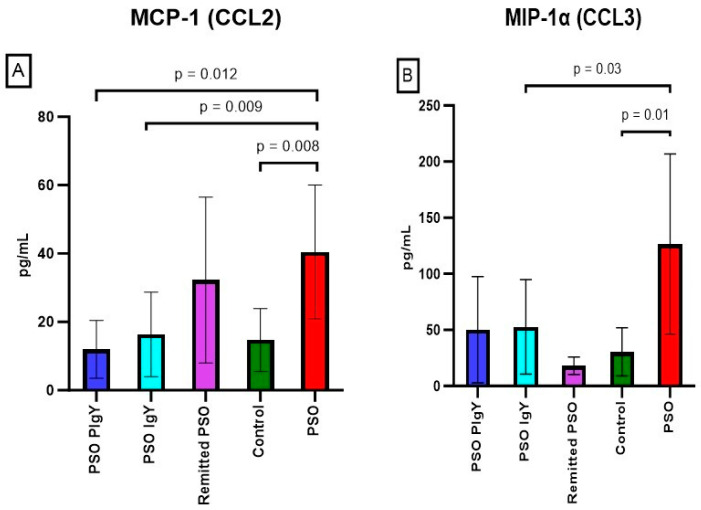
Serum levels of MCP-1/CCL2 and MIP-1α/CCL3. (**A**) MCP-1/CCL2 in the PSO PIgY group (12.0 ± 8.4), PSO IgY group (16.3 ± 12.4), remitted PSO group (32.2 ± 24.3), control (14.7 ± 9.2), and untreated PSO group (40.5 ± 19.6). (**B**) MIP-1α/CCL3 in the PSO PIgY group (50.2 ± 47.3), PSO IgY group (52.7 ± 42.2), remitted PSO group (18.1 ± 7.9), control (30.6 ± 21.4), and untreated PSO group (126.5 ± 80.4). The results are presented as the mean concentration values ± SD.

The analysis of chemokines in the serum of the PSO group revealed increased levels of MCP-1/CCL2 compared to those of the controls (40.5 ± 19.6 vs. 14.7 ± 9.2, respectively; *p* = 0.008). After IgY treatments (12.0 ± 8.4 for PSO PIgY and 16.3 ± 12.4 for PSO IgY), the serum values normalized (Figure 11A). Furthermore, the values for both treated groups were identical to the ones depicted in the control (*p* > 0.05), which underlines the normalization of these values after IgY treatment. For the naturally healed mice, we also obtained decreased mean values compared to those of the PSO group. Strong positive (r = 0.559) and negative (r = −0.842) correlations were obtained between MCP-1/CCL2 and IL-12 (p70) and IFN-γ, respectively, in IMQ mice (PSO group).

MIP-1α/CCL3 presented an elevated level in IMQ mice compared to that of the healthy mice (126.5 ± 80.4 vs. 30.6 ± 21.4, respectively; *p* = 0.01) and a tendency to normalize after IgY treatments (50.2 ± 47.3 for PSO PIgY and 52.7 ± 42.2 for PSO IgY) (Figure 11B). The values obtained for the IgY-treated groups and naturally remitted PSO group matched the ones obtained for the control group (*p* > 0.05). The serum levels of MIP-1α/CCL3 in the PSO group were strongly positively correlated to IL-6 (r = 0.794), IL-9 (r = 0.689), IL-10 (r = 0.925), and IL-13 (r = 0.624) values and negatively correlated to IL-15 (r = −0.621) levels. The analysis of MIP-1β/CCL4 serum levels revealed detectable values only for the PSO group (77.9 ± 49.6). A strong positive correlation (r = 0.745) was noticed between MIP-1β/CCL4 and MIP-1α/CCL3 in the PSO group. The serum levels of MIP-1β/CCL4 in the PSO group were also positively correlated to IL-6 (r = 0.572), IL-10 (r = 0.578), and IL-13 (r = 0.581) values and negatively correlated to IL-1α (r = −0.510) levels.

#### 3.3.7. Serum Levels of KC/CXCL1, IP-10/CXCL10, and MIG/CXCL9

**Figure 12:**
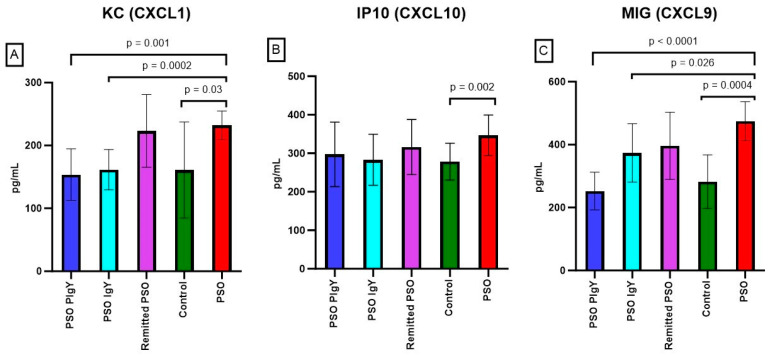
Serum levels of KC/CXCL1, IP-10/CXCL10, and MIG/CXCL9. (**A**) KC/CXCL1 in the PSO PIgY group (153.7 ± 41.0), PSO IgY group (161.4 ± 32.1), remitted PSO group (223.2 ± 57.8), control (161.0 ± 76.5), and untreated PSO group (232.0 ± 22.6). (**B**) IP-10/CXCL10 in the PSO PIgY group (297.1 ± 83.7), PSO IgY group (283.1 ± 66.3), remitted PSO group (316.1 ± 71.5), control (278.4 ± 47.6), and untreated PSO group (346.7 ± 52.6). (**C**) Expression of MIG/CXCL9 in the PSO PIgY group (252.2 ± 59.9), PSO IgY group (373.8 ± 92.9), remitted PSO group (396.3 ± 107.2), control (282.3 ± 85.5), and untreated PSO group (474.9 ± 61.9). The results are presented as the mean concentration values ± SD.

The analysis of KC/CXCL1 serum level revealed a normalization of the values in the IgY-treated groups (Figure 12A). As there were statistically significant differences between the PSO and control groups (232.0 ± 22.6 vs. 161.0 ± 76.5, respectively; *p* = 0.03), after the application of the IgY treatment, the values matched the ones obtained in the control group (153.7 ± 41.0 for the PSO PIgY group and 161.4 ± 32.1 for the PSO IgY group). For the naturally healed mice, the values of KC/CXCL1 remained elevated (223.2 ± 57.8), but the differences were not statistically significant when the values were compared to those of the control group. The serum levels of KC/CXCL1 in the PSO group were strongly positively correlated to IL-9 (r = 0.754), IL-10 (r = 0.823), IL-13 (r = 0.683), and MIP-1α/CCL3 (r = 0.730) values and negatively correlated with IL-15 (r = −0.708) levels. A positive correlation was also obtained between KC/CXCL1 and TNF-α (r = 0.361). The expression of IP-10/CXCL10 was also increased in IMQ mice compared to that of the control (346.7 ± 52.6 vs. 278.4 ± 47.6, respectively) and the differences between the experimental groups were statistically significant (*p* = 0.002) (Figure 12B). IgY treatments and natural healing led to lower levels (297.1 ± 83.7 for the PSO PIgY group, 283.1 ± 66.3 for the PSO IgY group, and 316.1 ± 71.5 for the remitted PSO group), and these levels were as low as the ones obtained in the control. The serum levels of IP-10/CXCL10 in the PSO group were strongly positively correlated to IL-9 (r = 0.920), IL-10 (r = 0.660), and MIP-1α/CCL3 (r = 0.528) values and negatively correlated to IL-15 (r = −0.590) and IL-17 (r = −0.591) levels.

The data obtained for the MIG/CXCL9 serum concentration revealed a significantly higher level in the PSO mice (474.9 ± 61.9, *p* = 0.0004) compared to that of the control and to the IgY-treated groups (*p* < 0.0001 for the PSO PIgY group and *p* = 0.026 for the PSO IgY group) (Figure 12C). A decreasing trend of the values was observed after IgY treatment (373.8 ± 92.9 for the PSO IgY group) and for the naturally healing group (396.3 ± 107.2). The normalization of the values was more obvious for the purified IgY-treated mice (252.2 ± 59.9), which is highlighted by the fact that the values perfectly match the ones obtained in the control group. The serum levels of MIG/CXCL9 in the PSO group were strongly positively correlated to IL-9 (r = 0.782) and IL-10 (r = 0.568) and negatively correlated to IL-12 (p70) (r = −0.637). A week positive correlation was also obtained between MIG/CXCL9 and IFN-γ (r = 0.331).

The statistical analysis revealed strong positive and negative correlations between these chemokines, both in the PSO and in the IgY-treated groups. Thus, in the PSO group, the KC/CXCL1 values were strongly positively correlated to IP-10/CXCL10 (r = 0.850) and MIG/CXCL9 (r = 0.893) values. IP-10/CXCL10 and MIG/CXCL9 values were strongly positively correlated (r = 0.923). After the purified IgY treatment, positive correlations were noticed between MIG/CXCL9 and KC/CXCL1 (r = 0.488) and IP-10/CXCL10 (r = 0.497), while KC/CXCL1 and IP-10/CXCL10 were negatively correlated (r = −0.459).

The overall statistical analysis of cytokine/chemokine values registered in the PSO PIgY and PSOIgY groups and compared to the naturally remitted PSO group showed that there are no differences in cytokine/chemokine levels between the IgY-treated groups and the spontaneously remitted PSO in this experimental model, as presented in Table 3.

### 3.4. Circulatory Immune Cell Populations

**Figure 13:**
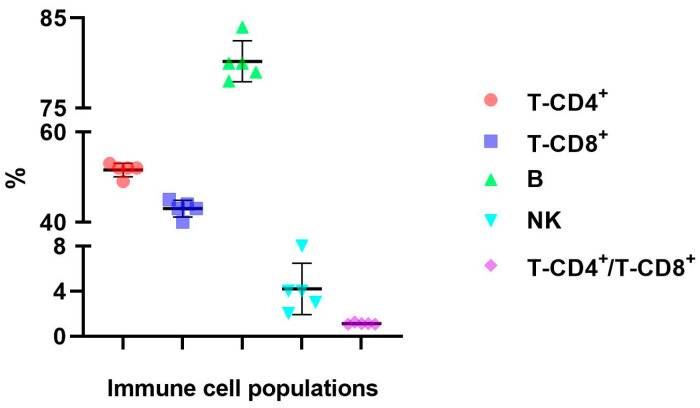
Distribution of the immune cell populations in the peripheral blood harvested from purified IgY-treated mice. Distribution of T-CD4^+^ (52 ± 1.4) and T-CD8^+^ (43 ± 1.9) lymphocyte subsets, B cells (80 ± 3.2), NK cells (4 ± 2.1), and T-CD4^+^/T-CD8^+^ ratio (1.20 ± 0.1) in the PSO PIgY group. The results are presented as mean values ± SD.

To fully investigate the immunological pattern of the PSO model and its IgY experimental therapy, we evaluated the effect of the NGC-purified IgY treatment on circulatory immune cell populations and subpopulations. Lymphocyte immunophenotyping from the peripheral blood was performed, and we identified and quantified various subpopulations, T-CD4^+^ and T-CD8^+^ lymphocyte subsets, B cells, and NK cells (Figure 12). The results were compared to the values obtained for the PSO IgY, naturally remitted PSO, control, and untreated PSO groups. The data revealed significant differences when the PSO PIgY group was compared to the untreated PSO group; differences were found for T-CD4^+^ (52 ± 1.4 vs. 47 ± 1.5, *p* = 0.0005), B cells (80 ± 3.2 vs. 31 ± 12.8, *p* = 2.2 × 10^−5^), and NK cells (4 ± 2.1 vs. 12 ± 3.3, *p* = 0.0006). For T-CD8^+^, the values were lower than those in the PSO group (43 ± 1.9 vs. 46 ± 2.8), but without statistical significance. After the NGC-purified IgY treatment, we observed the normalization of the values, hence the percentages of circulatory immune cells of PSO-PIgY group reaching the ones registered in the control (52 ± 1.4 vs. 53 ± 4.7 for T-CD4^+^, 43 ± 1.9 vs. 41 ± 1.8 for T-CD8^+^, 80 ± 3.2 vs. 85 ± 5.3 for B cells, 4 ± 2.1 vs. 4 ± 0.7 for NK cells, and 1.20 ± 0.1 vs. 1.29 ± 0.2 for the T-CD4^+^/T-CD8^+^ ratio). Immune cell circulatory populations were previously published by us in the present animal model [11], and in the present paper, we present only the data obtained for the NGC-purified IgY treatment (Figure 13).

In IMQ mice, we found several correlations between the immune cells’ values and cytokine/chemokine serum levels (Table 4).

## 4. Discussion

PSO is an autoimmune disease in which keratinocytes are prone to secrete various immune molecules linked to the disease’s immunopathogenesis [30], and their investigation can depict them as pathology and/or as therapy efficacy markers. We performed an experimental model of PSO that was previously reported by us and by other groups and we used IgY preparations as a possible future adjuvant therapy triggering gut microbiota in PSO. As the animal experimental model is not perfect and is actually an induced PSO model, we compared our experimental treatment with naturally remitted psoriasiform lesions.

We tested 32 cytokines/chemokines from the 40 documented cytokines/chemokines panel that were found to be involved in PSO pathogenesis. Of these, 18 cytokines/chemokines were found statistically significantly changed in the serum of the PSO animal experimental groups. Moreover, immune cells’ circulatory populations were investigated in relation to the serum cytokines/chemokines. The dynamics of immune parameters were followed to investigate the efficacy of the IgY therapy mirrored by immune-related biomarkers. As previously shown by us, the IgY therapy performed in the psoriatic dermatitis animal model reduced the time of PSO lesion healing by 2–3 days, which in terms of overall mice age is significant [11,24]. Moreover, the cumulative PASI score in the naturally remitted group did not drop to the score of the control group, as it did in the therapy group. The latter can also explain some of the remanence of immune-related inflammatory parameters that are further discussed in the naturally remitted group.

In this section, we discuss our findings regarding the involvement of pro- and anti-inflammatory cytokines/chemokines, grouped by their action and regardless their origin, namely, secreted by immune or non-immune cells. Moreover, we discuss their relationship between immune cell populations and the molecules that sustain cells’ cross-talk.

### 4.1. Inflammatory Cytokines’ Improvement after IgY Treatment in Experimental PSO Model

Within the skin’s microenvironment, IL-12 and IL-6 are important regulators of Th1/Th2 differentiation: IL-12 induces the differentiation of naïve T cells into Th1 cells, while IL-6 promotes the IL-4-dependent induction of Th2 differentiation and inhibits Th1 polarization [31,32]. IL-6 is correspondingly involved in the differentiation of Th17 cells in the presence of IL-23 [33]. 

Similarly to the previous findings in humans and in mouse models [34,35,36,37,38], in our study, the values of IL-12p70 were up to 10 times higher in the PSO-induced mice compared to those of the control and/or IgY-treated mice. Notably, in terms of IgY’s therapeutic effect upon inflammatory cytokines, both tested compounds of IgY had a similar effect upon IL-12 circulatory concentration, with a slight improvement in the purified IgY.

Our results reveal a high serum level of IL-6 in the PSO-induced mice compared to the controls, matching the previously reported elevation of this cytokine in human and in experimental models [39,40]. IL-6 serum level in the PSO group was found to be eight times higher when compared to the IgY-treated groups. The normalization of IL-6 values identified by us is more obvious in IgY-treated groups than in naturally remitted mice. Interestingly, in the naturally healing group, IL-6 values continue to be increased, even after the normal remission of the PSO lesions, which suggests IL-6 is a marker of efficient therapy [41,42]. We also obtained a strong negative correlation between IL-6 and IL-12 (p70) in the PSO group that is in accordance with the processes triggered by cells upon IL-6 or IL-12 regulation.

In PSO, IL-12 induces Th1 cells, which secretes pro-inflammatory cytokines, including IL-2, TNF-α, and IFN-γ. These pro-inflammatory cytokines, together with IL-17 and IL-22, activate keratinocytes that produce mainly pro-inflammatory cytokines/chemokines, thus contributing to the perpetuation of the inflammatory status in PSO [43,44,45].

Our obtained data on the circulatory levels of TNF-α and IFN-γ revealed highly increased levels in the PSO mice. After IgY treatments, we observed the normalization of these values, especially for mice treated with purified IgY, where the circulatory level of this cytokine dropped to the control values. The analysis of the serum IFN-γ level revealed detectable concentrations only for the IMQ mice. It is possible that the multiplex method used to identify circulatory levels can have its limitations; thus, reduced levels of IFN- γ could not be detected in our workflow. We found a strong negative correlation between IFN-γ and IL-12 (p70) in the PSO group that can be explained by the predominance of other Th1-specific cytokines, e.g., TNF-α, in the psoriatic inflammatory events.

Investigating the IL-1 main members, we obtained, namely, increased IL-1β, while decreased IL-1α similar to previously reported results [46]. Significant decreased mean values were found by us for IL-1α in the IMQ mice compared to the controls, values that were strongly positively correlated to TNF-α values and negatively correlated to IL-1β. After IgY treatments or the natural healing of psoriatic lesions, we observed a pronounced upward trend of IL-1α for all experimental groups. Thus, IgY treatments have led to the normalization of IL-1α serum values, matching the control values. The serum levels of IL-1β were found highly increased in the IMQ mice (PSO group) and, after IgY treatments, we observed the normalization of these values, especially for the IgY group, where the circulatory level of these cytokines was similar to that of the controls. For the natural healing group, the values of IL-1β were also found decreased at the end of experiment, but there are still statistically significant differences compared to those of the controls, suggesting that this cytokine has a higher remanence in circulation and/or the cells that secrete it remain active. We obtained a strong positive correlation between IL-1β and IL-12 (p70) in the PSO group and also in the purified IgY-treated group. This finding suggests that pro-inflammatory cytokines have a strong synergic action and that, even in the IgY-treated group, these cytokines still are active, probably in the normal regenerative process of the skin. Moreover, the correlation can be as well explained by macrophages’ plasticity in inflammation and the polarization towards the M1 population that induces both high IL-1β and IL-12 secretion [47]. As shown above for IL-12 and IFN-γ, we also obtained a strong negative correlation between IL-1β and IFN-γ in the PSO mice. The high inflammatory status induced by PSO counterbalances these two cytokines, a balance that is regulated by mTORC2 activation as recently reported [48]. Probably, other cytokines from the pro-inflammatory arsenal counterbalance IFN-γ, but this cytokine was detectable only in the PSO group.

IL-9 belongs to the IL-2 cytokine family and has pleiotropic immune functions. In our study, IL-9 presented significant elevated levels in the IMQ mice compared to that of the control, while being negatively correlated with IL-15. As they are structurally similar and belong to a large family comprising several other cytokines, their functions compensate each other within PSO pathogenesis [49,50,51]. Nevertheless, a decreasing trend was observed after IgY treatments, although IL-9 values remained significantly higher compared to those of the controls. For the naturally healed mice, the mean values of serum IL-9 were similar to those of the control. The serum levels of IL-9 in the PSO group were also strongly positively correlated to IL-6 and negatively correlated to IL-12 levels. These complex immune cross-talks in the cytokine network still have to be deciphered and IL-9 remains an understudied cytokine, although it is involved in many immune/biological functions.

IL-15 and its receptor are overexpressed in PSO and seem to play an early role in PSO pathogenesis [52]. Our data showed significantly elevated IL-15 serum levels in the IMQ mice compared to those of the control, and significant decreases after IgY treatments and natural healing, results that match those previously reported by Villadsen et al. in a xenograft mouse model treated by blocking IL-15 biological activity [52]. We noticed a strong positive correlation between IL-15 and IFN-γ in the IMQ-treated mice. As already known, the obtained correlation is due to the fact that IL-15 induces the production of other proinflammatory cytokines (e.g., TNF-α, IFN-γ, and IL-17) [53].

The most important member of the IL-17 family is IL-17A, highly involved in PSO pathogenesis [54]. Increased levels of Th17 cells and IL-17A have been previously reported in psoriatic lesions and in the peripheral blood of PSO patients [55], parameters that are positively correlated with the PASI score [51]. In our study, the IL-17 circulatory level was higher in the IMQ mice compared to that of the control and its levels lowered after IgY treatment or natural healing. The analysis of IL-17 serum levels revealed statistically significant differences between the males from the PSO group and the control males, differences that were not observed in females nor in other experimental groups; these gender differences in immune-related molecules were previously reported by us in other skin pathologies [56] and in autoimmune diseases [57]. The serum levels of IL-17 in the PSO group were strongly positively correlated with the IL-15 level, a correlation that matches the previously reported data in other autoimmune diseases, like rheumatoid arthritis [58].

### 4.2. Anti-Inflammatory Cytokines’ Improvement after IgY Treatment in the Experimental PSO Model

Th2-specific cytokines (anti-inflammatory cytokines) have been less studied in PSO compared to the Th1/Th17 profile. Contradictory results were reported regarding the anti-inflammatory cytokines levels in PSO [45,59]. In our study, IL-5 and IL-10 serum levels had elevated values in the PSO group, while IL-13 showed significantly decreased values in the IMQ mice compared to that of the control. IgY treatments normalized the secretion of these cytokines, and the IL-10 levels were similar to those of the control. Even though the serum level of IL-5 decreased significantly after IgY treatments, the values still remained higher compared to those of the control. For the naturally healing group, we also obtained decreased mean values, but the normalization of the values was more obvious in the case of IgY treatments. The serum level of IL-13, found decreased in the IMQ mice, remained low after both IgY treatments and the remission of induced PSO lesions. A strong positive correlation was found between IL-13 and IL-10, and a negative one between IL-13 and IL-1β in the IMQ mice. These correlations prove that there is an intrinsic balance between Th1 and Th2 cytokine’s profiles.

### 4.3. Inflammatory Chemokines’ Improvement after IgY Treatment in the Experimental PSO Model

In accordance with previous studies regarding chemokine involvement in PSO [60,61,62], our data showed an elevated level of MCP-1/CCL2 in the IMQ mice compared to that of the healthy mice. We also observed the normalization of the values after IgY treatments. In the case of the purified IgY treatment, the MCP-1/CCL2 serum level dropped to the control’s ones. In the PSO group, we obtained a strong positive correlation between MCP-1/CCL2 and IL-12 (p70), and a strong negative correlation with IFN-γ. These findings are in accordance with the MCP-1 and IL-12 synergic action that enhance the recruitment and activation of innate immune cells [63]. 

In our experimental model, we obtained elevated serum levels for MIP-1α/CCL3 in the IMQ mice and a tendency to normalize after IgY treatments. The analysis of MIP-1β/CCL4 revealed detectable values only for the PSO group. MIP-1α/CCL3 and MIP-1β/CCL4 values were positively correlated to T-CD3^+^-circulating lymphocytes in the PSO mice. 

Our results show elevated serum levels for CXCL1, and 10 and 9 chemokines in IMQ mice as compared to those of the control, matching the results previousy obtained in PSO patients [64]. A decreasing trend of the values was observed after IgY treatments or natural remission. The analysis of KC/CXCL1 serum level revealed a normalization of the values in the IgY-treated groups, while for the naturally healed mice, the values still remained elevated. In the case of IP-10/CXCL10, IgY treatments and natural remission also led to a lower serum level that was statistically identical to the control levels. For MIG/CXCL9, the normalization of the values was more obvious in the purified IgY-treated mice compared to those of the other groups. Similar to human studies, in our PSO group, the KC/CXCL1 circulatory values were found positively correlated to TNF-α values, while MIG/CXCL9 values were positively correlated to IFN-γ, correlations that prove the existence of a cytokines–chemokines loop where interferons and TNF activate the chemokine release and the chemokines further induce cytokine production [65].

In our experimental model, TNF-α, IL-6, and IL-1β cytokines had extremely high circulatory levels in PSO. Upon experimental therapy with IgY (standard or purified), the serum values regained their lower values in the range of naturally remitted skin inflammation and/or compared to the healthy mice.

### 4.4. Normalization of Circulatory Immune Cells after IgY Treatment in the Experimental PSO Model

The circulatory levels of immune cell populations were found normalized after NGC-purified IgY therapy, similar to IgY compound treatment previously published by us [24]. Specifically, the T-CD4^+^ and T-CD8a^+^ lymphocyte subsets, B-CD19^+^ and NK1.1^+^ cells, regained their normal values upon treatment. As expected, the T-CD4^+^/T-CD8a^+^ ratio in the peripheral blood showed the normalization pattern of the IgY-treated PSO. The correlation between immune cells and cytokine/chemokine levels was found both negative and positive; part of this correlation has been previously published in the literature, while another part still needs in depth evaluation to establish the intimate mechanisms and clinical significance.

Overall, the best cytokine/chemokine correlation was found between T-CD4^+^ and IL-17 (r = 0.782), but only for the males from the PSO group. Moreover, T-CD4^+^ values were strongly positively correlated to IL-6 and IL-9 serum levels and negatively correlated to IL-13 level. 

Even though IFN-γ was weekly positively correlated with T-CD8^+^ and NK cells, its role in the PSO experimental model seems not to be essential in comparison to the other cytokines. This assertion is sustained also by the contradictory results obtained for IFN-γ, aberrantly correlated with IL-12, IL-1-b, and MCP-1. It is clear that the psoriatic inflammatory events are governed by the predominance of other Th1-specific cytokines, e.g., TNF-α and IL-12, for which we obtained high circulatory levels that decreased significantly after IgY therapy.

We can highlight some study limitations for the present work. The IMQ PSO model is a standard one, used by various groups; therefore, all the histopathological parameters seen in PSO patients can be reproduced in this animal model, including immune-related parameters [24,27,66,67]. However, we do not deny that this model does not perfectly reproduce human PSO, where autoimmune antigen/mechanisms trigger this disease and probably genetically manipulated strains could better reproduce the disease [68,69]. This IMQ PSO animal model actually reproduces the acute phase of the disease, that is, other clinical parameters characterized by high inflammation status that is depicted at both lesion and systemic levels [69]. This is the reason why, in our study, we also investigated a group of induced PSO that was left to heal naturally and compared their immune parameters with the other investigated groups. After healing naturally, some of the investigated immune parameters reached the control levels, but others were still elevated, which explains why even a 6-day IMQ induction of the psoriatic dermatitis in this mice strain can induce longer-term immune deregulations. To our knowledge, these are the first immune-related results on naturally remitted IMQ PSO in C57Black mice. We do not exclude that, in other IMQ-induced PSO mouse strains, the obtained immune-related parameters can differ, so that each model may have its own characteristics. 

Another limitation of the study relates to the multiplex method used to identify circulatory levels. Therefore, reduced levels of cytokines/chemokines (in our case, IFN-γ) could not be detected and, hence, some of the cytokine/chemokine levels and further alterations could have been concealed in our investigation. The oral ingestion of IgY preparations still has to be studied in various designs so as to be further implemented as an adjuvant therapy in humans and, therefore, represents another limitation of our study. In rodents and ruminants, IgY is functionally stable and resists the enzymes within the gastro-intestinal tract passage; however, in humans, this aspect should be thoroughly studied.

Complex studies regarding the cytokine/chemokine immunological profile and immune cell profiling in terms of therapeutical approaches targeting the gut microbiota that can improve PSO development still are lacking. In recent years, the link between the gut microbiota and PSO initiation and development has been shown [3,70,71,72,73,74].

Various interventions upon the gut microbiota have shown that, if microbiota normalization is obtained, cytokine normalization reduces psoriatic inflammation. Similar to our findings, the cytokine normalization upon gut intervention was accompanied by psoriatic lesions’ alleviation [75,76,77]. 

The gut–skin axis immune link in PSO is lately opening new research avenues and, in this complex relationship, there are still mechanisms to be discovered.

## 5. Conclusions

In a PSO mouse model, the healing effect of psoriatic lesions were followed using IgY therapy targeting the gut microbiota. The healing effect was investigated macroscopically, histologically, and by evaluating the immune parameters involved in PSO pathogenesis: circulatory immune cell and cytokine/chemokine serum levels. The healing process was more efficient in the IgY-treated group compared to the naturally healing group and was statistically sustained by an increased normalization of the investigated parameters. The cellular immune parameters regained their normal values after IgY therapy, particularly for T-CD4^+^ and B and NK cells, especially, for NGC-purified IgY. As cytokines are crucial molecules for maintaining lymphocyte homeostasis, from the tested types of immune molecules, half experienced the normalization of their levels upon IgY therapy. Upon experimental therapy with IgY (standard/purified), the cytokine serum values were not different between the IgY-treated groups and the spontaneously remitted PSO in this experimental model. These findings suggest the need for future thorough studies on the intimate mechanism governing the gut microbiota that influences skin-related auto-immune reactions.

## Figures and Tables

**Table 1:** Concentrations of the protein fractions corresponding to peaks 1–4 after IgY was subjected to NGC purification.

Peak Number	Peak 1	Peak 2	Peak 3	Peak 4
Concentration (µg/mL)	38	125	39	28

**Table 2:** PASI score comparison between the IgY-treated vs. naturally remitted PSO groups.

Day	Day 1–9	Day 10	Day 11	Day 12	Day 13	Day 14
*p*-values for the IgY-treated vs. naturally remitted PSO groups	*p* > 0.05	*p* = 0.069	*p* = 0.003	*p* = 0.0019	*p* = 1.2 × 10^−4^	*p* = 1.56 × 10^−4^
		Day 16	Day 17	Day 18	Day 19	Day 20
		*p* = 1.1 × 10^−5^	*p* = 1.8 × 10^−6^	*p* = 1.1 × 10^−6^	*p* = 1.8 × 10^−6^	*p* = 1.6 × 10^−7^

**Table 3:** Comparison of cytokine/chemokine values among the PSO PIgY, PSO IgY, and naturally remitted PSO groups (*p*-values).

Cytokine/Chemokine	IL-12 (p70)	IL-6	TNF-α	IL-1α	IL-1β
PSO PIgY *p*-values	0.187	0.400	0.767	0.470	0.940
PSO IgY *p*-values	0.270	0.275	0.910	0.696	0.943
Cytokine/chemokine	IL-9	IL-15	IL-5	IL-10	MCP-1
PSO PIgY	0.695	0.999	0.459	0.996	0.337
PSO IgY	0.169	0.997	0.505	0.999	0.487
Cytokine/chemokine	MIP-1α	KC	IP-10	MIG	
PSO PIgY	0.901	0.301	0.993	0.142	
PSO IgY	0.835	0.327	0.933	0.993	

**Table 4:** Correlations between circulatory immune cells and the serum level of cytokines/chemokines in IMQ mice (r-values).

Immune Cells/ Cytokines/ Chemokines	T-CD3^+^	T-CD4^+^	T-CD8^+^	B	NK
IL-1α	NS	NS	NS	r = 0.620	NS
IL-1 β	NS	r = −0.468	r = 0.456	r = −0.743	NS
IFN-γ	r = 0.32	NS	r = 0.332	NS	r = 0.34
IL-5	r = −0.749	NS	NS	NS	r = −0.584
IL-6	r = 0.552	r = 0.557	NS	NS	r = 0.470
IL-9	NS	r = 0.405	r = −0.602	NS	NS
IL-10	NS	NS	NS	r = −0.491	NS
IL-13	NS	r = −0.430	NS	r = −0.869	NS
IL-17	NS	r = 0.782	r = 0.546	r = −0.486	NS
TFN-α	r = −0.655	NS	NS	r = 0.524	NS
MIP-1α	r = 0.407	NS	NS	NS	r = 0.525
MIP-1β	r = 0.652	NS	NS	NS	r = 0.468

NS = not statistically significant.

## Data Availability

The datasets used and/or analyzed during the present study are available from the corresponding author upon reasonable request.
